# Fabrication and characterization of reversible thermochromic wood veneers

**DOI:** 10.1038/s41598-017-17238-9

**Published:** 2017-12-05

**Authors:** Xiaodong Zhu, Yu Liu, Ningwen Dong, Zhao Li

**Affiliations:** 1 Key Laboratory of Bio-Based Material Science and Technology (Ministry of Education), Northeast Forestry University, Harbin, Heilongjiang 150040 China; 2 College of Materials and Engineering, Northeast Forestry University, Harbin, Heilongjiang 150040 China

## Abstract

Leuco dyes are widely used as functional materials for their thermosensitive chromogenic nature. The influences of thermochromic compounds and impregnation processing conditions on thermochromic wood veneer properties were investigated in this paper. The thermochromic compounds included thermochromic dye (ODB-2), color developer (bisphenol A) and solvent (1-tetradecanol). To achieve the maximum color change, the optimum mixing ratio of ODB-2 to bisphenol A to 1-tetradecanol was 1:2:60. Juglans mandshurica veneers were ultrasonically impregnated with reversible thermochromic compound suspensions. Analysis of variance showed that the influences of impregnation parameters on veneer color change were significant at the 0.05 level. The optimum thermochromic wood veneer can be obtained by impregnating with a thermochromic compound suspension for 2.0 min at 65 °C. In this paper, the thermochromic properties of wood veneers were evaluated, and functional thermochromic veneers exhibited excellent properties and thermostability.

## Introduction

Wood color plays an important role in the commercialization process^1^ and is dependent on species, tree genetic resources, drying process and thermal treatments^2–5^. To improve surface quality and visual characteristics, wood dying has become an important technology to functionalize wood materials. The development of reversible thermochromic materials has received the most attention in recent years^6–10^. Color changing features can enrich the decorative effect of wood materials and can be used as temperature indicators.

Reversible thermochromic composites, consisting of leuco dye, color developer and organic solvent, are characterized by coloration in the solid state and discoloration in the molten state^11^. The color changes due to structural modifications occur with temperature. Fluoran leuco dyes are widely used in fields of engineering drawings, biochemical analysis, light-emitting probes, invoices, and printing inks^12–15^ and show good sensitivity and thermochromic stability.

The classical example of a suitable dye is the ionochromic crystal violet lactone. Liu *et al*. colored poplar veneer by ultrasonic impregnation using crystal violet lactone, biphenyl A, 1-tetradecanol and sodium thiosulfate. The maximum ΔE of poplar veneer was obtained with the optimum mixing ratio of 1:8:50:1^16,17^. Jiang *et al*. analyzed the relationship between thermochromic properties and solvent melting points, and thermochromic wood with a lower temperature response was obtained with 1-dodecanol^18^. Hu *et al*. prepared a photochromic wood material by adding microcapsules into wood fibers and wood coatings, and the wood altered its appearance from a veneer color to a blue color when exposed to sunlight^19,20^. Relevant studies on problems in thermochromic agent preparation, the manufacturing technology and thermochromic performance evaluation are ongoing.

Indeed, high-grade wood products with nature appearance in color and texture are becoming more and more popular, some traditional dyes are sufficiently sensitive to heat but would not currently be acceptable. The dye 2-anilino-6-(dibutylamino)-3-methyl fluoran (ODB-2) is widely used in thermo-paper manufacturing. Previous studies showed that the interaction between solvent, developer and ODB leuco dyes had significant effects on reversible thermochromic performances^21,22^. Yang *et al*. optimized the ratio of thermosensitive black, bisphenol A and hexadecanol for the reversible thermochromic composition as 1:2:50^23^. In this paper, thermochromic compounds consisting of ODB-2 dye, bisphenol-A developer and 1-tetradecanol organic solvent were used for veneer impregnation. The effects of synthesis conditions on the thermochromic properties of wood veneers were studied. In addition, fourier transform infrared spectroscopy (FTIR) analyses were applied to investigate the thermochromic mechanism of wood veneer.

## Results and Discussion

### Determination of the optimum thermochromic composition formula

In this study, the orthogonal design was used to optimize thermochromic composition formula to obtain best color change, and ΔE values were calculated to describe the degree of color change. The colorimetric parameters of thermochromic compounds were obtained by measuring the surfaces of glass plates coated with thermochromic compounds. The color parameters of coated glass plates were measured at 0 °C and 70 °C. The total color difference values ΔE of the thermochromic compounds were used to select the optimum processing parameters. The test results are shown in Table 1.

**Table 1:** Results and range analysis of thermochromic compound experiments.

Test	Mixing ratio of ODB-2 and bisphenol	Interaction	Mixing ratio of ODB-2 and 1-tetradecanol	Null	ΔE
1	1(1:1)	1	1(1:20)	1	27.39
2	1(1:1)	2	2(1:40)	2	27.67
3	1(1:1)	3	3(1:60)	3	27.09
4	1(1:1)	4	4(1:80)	4	29.64
5	2(1:2)	1	2(1:40)	3	30.59
6	2(1:2)	2	1(1:20)	4	31.94
7	2(1:2)	3	4(1:80)	1	29.18
8	2(1:2)	4	3(1:60)	2	32.32
9	3(1:3)	1	3(1:60)	4	28.80
10	3(1:3)	2	4(1:80)	3	30.59
11	3(1:3)	3	1(1:20)	2	29.03
12	3(1:3)	4	2(1:40)	1	31.33
13	4(1:4)	1	4(1:80)	2	25.32
14	4(1:4)	2	3(1:60)	1	27.86
15	4(1:4)	3	2(1:40)	4	28.62
16	4(1:4)	4	1(1:20)	3	30.84

The color difference value ΔE indicates the color changes of the thermochromic material before and after temperature variation. As shown in Table 1, the ΔE values of different thermochromic compounds ranged from 25.32 to 32.32. The relationships of ΔE and individuals’ vision (Table 2) indicate that all thermochromic compounds had good thermochromic performances. As the temperature varied from 0 °C to 70 °C, the color change was obvious and could be easily noticed by vision.

**Table 2:** Relationship between ΔE and vision.

Color-change value ΔE	Vision
0–0.5	Unnoticeable change
0.5–1.5	Slight change
1.5–3.0	Appreciable change
3.0–6.0	Recognizable change
6.0–12.0	Obvious change
Over 12.0	Very obvious change

Range analysis were performed and the results presented in Table 3 indicate the average ΔE value of each factor at various levels, the main effect factor on ΔE is the mixing ratio of ODB-2 and bisphenol A, followed by the mixing ratio of ODB-2 and 1-tetradecanol.

**Table 3:** Intuitive analysis of test results.

Index	Mixing ratio of ODB-2 and bisphenol	Interaction	Mixing ratio of ODB-2 and 1-tetradecanol
K1	27.9500	28.0250	29.8000
K2	31.0075	29.5150	29.5600
K3	29.9375	28.4800	29.0175
K4	28.1600	31.0325	28.6825
Range	3.0575	3.0075	1.1175

RankingMixing ratio of ODB-2 and bisphenol > Mixing ratio of ODB-2 and 1-tetradecanol

K_i_-average values for level i = 1,2,3,4; Range = K_max_-K_min_, larger range value indicating more significant effects.

When the mixing ratio of ODB-2 and bisphenol A increased to 1:2, the ΔE value increased in an obvious manner, it is due to the reaction of ODB-2 and bisphenol A. As an electron donor, increasing the amount of bisphenol A induced more contact with leuco agent ODB-2. When the temperature was varied from 0–70 °C, the leuco effect was enhanced, and the ΔE value increased. As the mix ratio of ODB-2 and bisphenol A increased to 1:3 and 1:4, the ΔE value displayed a decreasing tendency. The excessive bisphenol A was dissolved in 1-tetradecanol and was used to diluted the solution.

The range analysis results of ODB-2 and 1-tetradecanol mixing ratio showed that the ΔE value decreased with the increase in 1-tetradecanol. Rarely, small amounts of 1-tetradecanol can dissolve and disperse ODB-2 and bisphenol A. As the dosage of 1-tetradecanol increases, it can significantly promote the reaction between ODB-2 and bisphenol A. However, the addition of 1-tetradecanol can also dilute the solution, explaining the decrease in the ΔE value.

**Figure 1:**
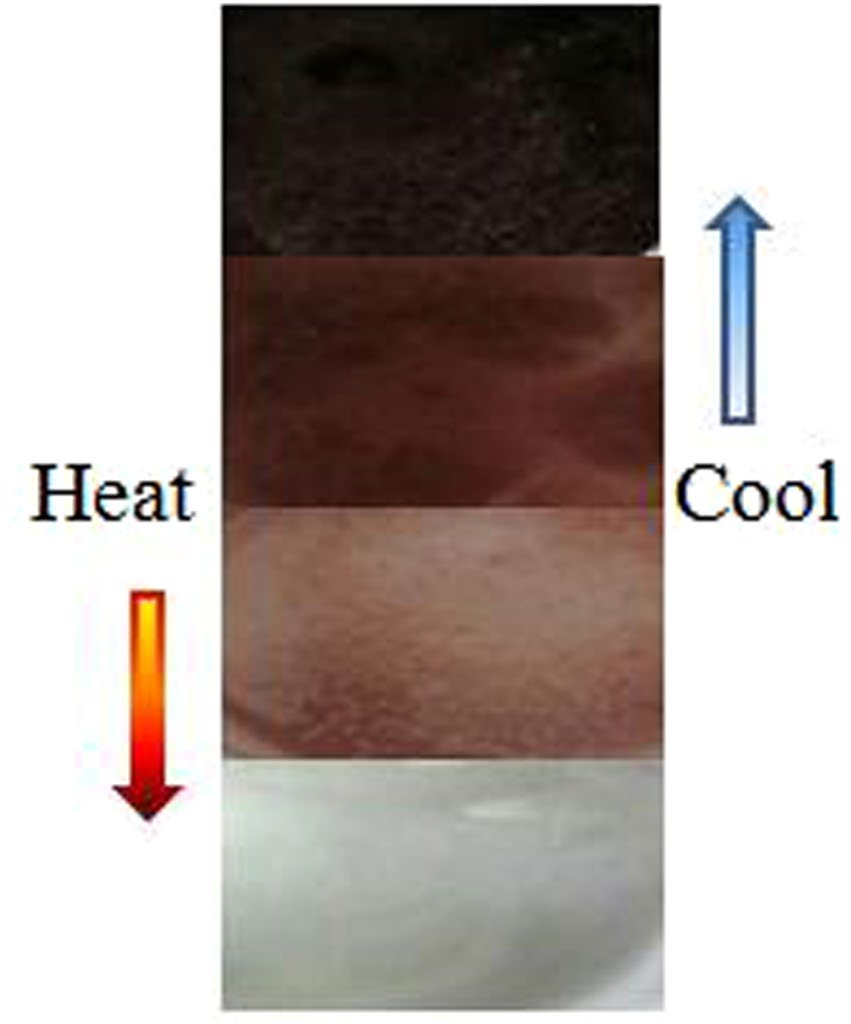
Color changes of the thermochromic compound.

The corresponding analysis of variance (ANOVA) observed that mixing ratio of ODB-2 and bisphenol act as significant factor for ΔE value (*p* < 0.05), while the interaction was also significant for ΔE value (Table 4). The optimum ratio of ODB-2 to bisphenol A to 1-tetradecanol was 1:2:60. Figure 1 shows the color changes of the thermochromic compound synthesized under the optimum ratio from 0 °C to 70 °C.

**Table 4:** Results of ANOVA.

Source	Sum of squares	df	Mean square	F	Sig.
Mixing ratio of ODB-2 and bisphenol	25.782	3	8.594	9.067	0.012
Interaction	21.361	3	7.120	7.513	0.019
Mixing ratio of ODB-2 and 1-tetradecanol	3.078	3	1.026	1.082	0.425
Error	5.687	6	0.948		
Total	55.907	15

**Figure 2:**
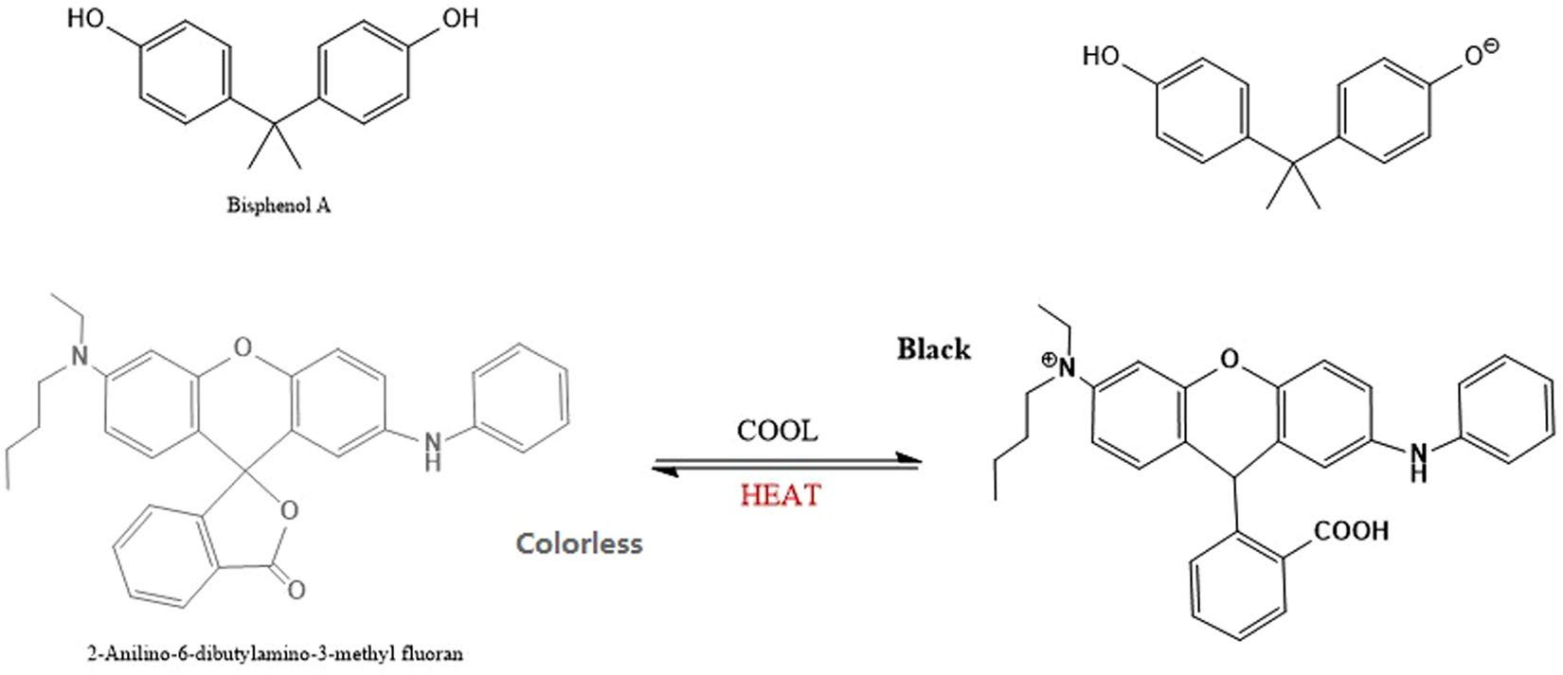
Thermochromic response mechanism of the ODB-bisphenol A system.

The mechanism of color transition induced by the ODB-bisphenol A interaction is shown in Fig. 2. In the thermochromic compound system, the color former (ODB-2) is an electron-donating compound, and the color developer (bisphenol-A) acts as a proton donor to induce the colored stated of leuco dye components. When the lactone ring of the color former is closed, the thermochromic compound is in its colorless state. The addition of a proton induces the ring open, leaving the dye is in its color state^24^.

### Optimum fabrication process for reversible thermochromic wood veneers

Based on the optimum thermochromic compound formula, the effects of wood veneer impregnation temperature and time on the ΔE values of thermochromic wood veneers were investigated. Larger ΔE values indicated better thermochromic performance. Range and ANOVA analyses were used to determine the optimum impregnation parameters.

**Figure 3:**
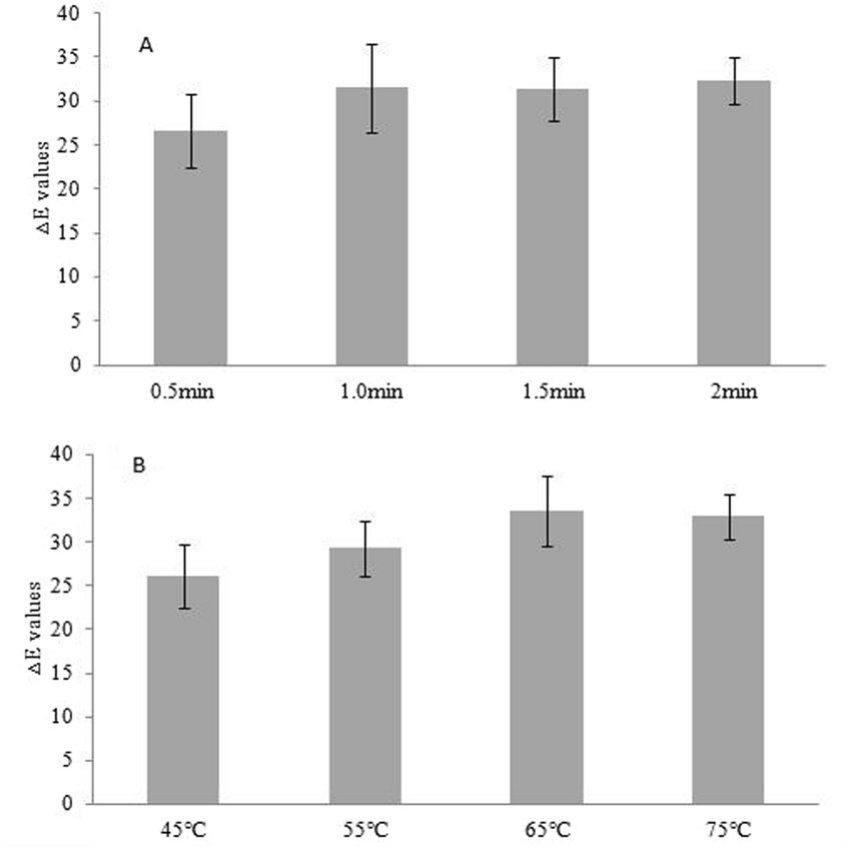
Effects of thermochromic wood veneer processing parameters (**A**) impregnation time, (**B**) impregnation temperature) on ΔE values. Values are given as the mean ± s.d.

As shown in Table 5, the mean ΔE values of the thermochromic wood veneers ranged from 20.77 to 37.96. All samples showed good thermochromic performances. From the range analysis of the orthogonal experiment (Fig. 3), the influential degree of the two factors was impregnation temperature > impregnation time. As the impregnation temperature increased from 45 °C to 65 °C, the ΔE values significantly increased, and the color variations were correlated with the amount of thermochromic compounds in the wood veneers. In the ultrasonic impregnation process, the use of the ultrasonic technique increased the thermochromic compound’s vibration displacement, velocity, molecular dispersion and energy^25^, thereby increasing the mass transfer of thermochromic compounds impregnated into the wood veneers. Meanwhile, the cavitations generated during ultrasonic impregnation promoted the diffusion of thermochromic compounds into the wood veneers. As Fig. 3 showed that the maximum ΔE values were obtained at the impregnation temperature level of 65 °C.

**Table 5:** Results and range analysis of the impregnated process for thermochromic wood veneers.

Test	Impregnation temperature	Interaction	Impregnation time	Null	ΔE			
					І	II	III	Mean
1	1(45 °C)	1	1(0.5 min)	1	20.77	23.13	22.26	22.05
2	1(45 °C)	2	2(1.0 min)	2	20.93	24.9	26.07	23.97
3	1(45 °C)	3	3(1.5 min)	3	27.87	28.44	28.04	28.12
4	1(45 °C)	4	4(2.0 min)	4	29.89	30.43	30.19	30.17
5	2(55 °C)	1	2(1.0 min)	3	31.14	30.42	31.89	31.15
6	2(55 °C)	2	1(0.5 min)	4	25.34	24.62	23.92	24.62
7	2(55 °C)	3	4(2.0 min)	1	32.3	33.28	30.74	32.10
8	2(55 °C)	4	3(1.5 min)	2	30.05	27.92	30.03	29.33
9	3(65 °C)	1	3(1.5 min)	4	37.96	35.95	34.24	36.05
10	3(65 °C)	2	4(2.0 min)	3	36.73	37.64	34.46	36.28
11	3(65 °C)	3	1(0.5 min)	2	27.42	28.83	26.17	27.47
12	3(65 °C)	4	2(1.0 min)	1	34.83	34.51	34.22	34.52
13	4(75 °C)	1	4(2.0 min)	2	30.98	31.40	30.44	30.94
14	4(75 °C)	2	3(1.5 min)	1	35.81	29.84	30.48	32.04
15	4(75 °C)	3	2(1.0 min)	4	35.21	37.55	36.2	36.32
16	4(75 °C)	4	1(0.5 min)	3	33.08	30.95	33.12	32.38

The impregnation time factor also had a significant effect on the ΔE values of the thermochromic wood veneers. As the impregnation time increased, the ΔE values gradually increased. During the wood impregnation process, the thermochromic compounds adsorbed on the wood surface first and then penetrated into the interior of the wood through the cell cavity and cell gap. As the impregnation time increased, the thermochromic compounds could become fully immersed into the wood veneers, which resulting in increases in the ΔE values.

Within the limits of the experimental conditions, the optimum thermochromic wood veneer can be fabricated by impregnation of the veneer with the thermochromic compounds (with a 1:2:60 ratio of ODB-2 to bisphenol A to 1-tetradecanol) suspension for 2.0 min at 65 °C, since K_i_ was highest at this combinations.

ANOVA was used to evaluate the significance of impregnation processing factors on ΔE values at α = 0.05 level. The results of ANOVA presented in Table 6, indicated that impregnation temperature and time had significant effects on the ΔE values of thermochromic wood veneers.

**Table 6:** Analysis of variance test results for the ΔE values of thermochromic wood veneers.

Source	Sum of squares	df	Mean square	F	Sig.
Impregnation temperature	436.120	3	145.373	25.548	0.000
Interaction	39.426	3	13.142	2.310	0.092
Impregnation time	242.565	3	80.855	14.210	0.000
Error	216.223	38	5.690		
Total	934.335	47			

### Properties of thermochromic wood veneers

**Figure 4:**
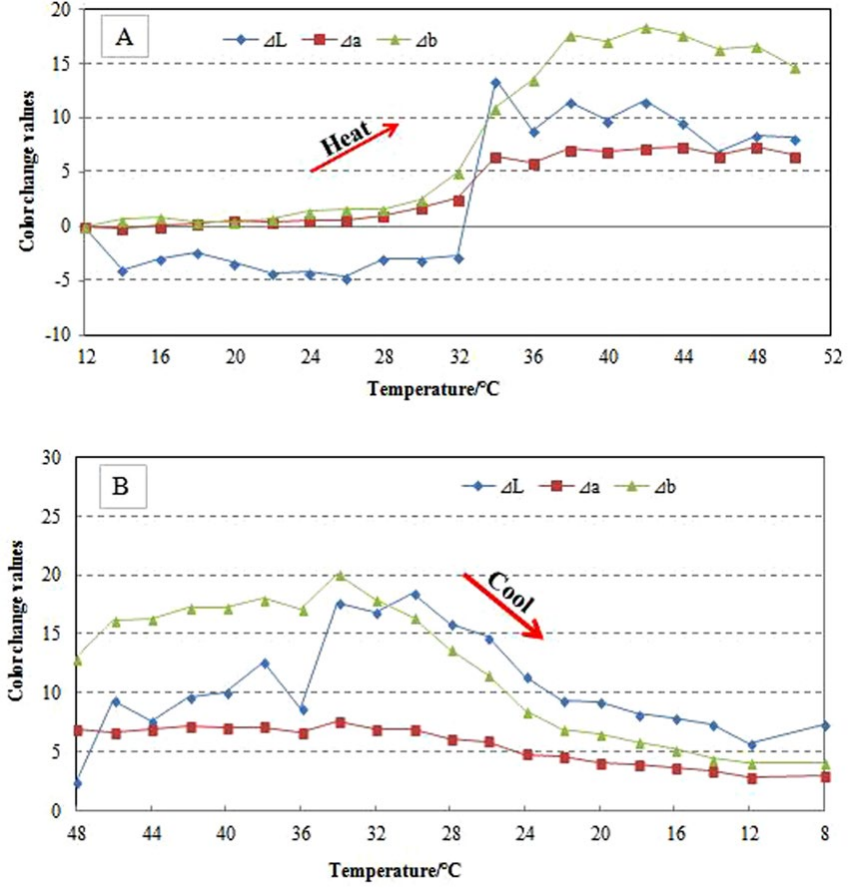
Color change values of thermochromic wood veneers during the heating (**A**) and cooling (**B**) processes.

The colorimetric values of thermochromic wood veneers were measured during heating from 12 °C up to 50 °C and cooling down to 12 °C. The color changes were calculated, and the results are shown in Fig. 4.

**Figure 5:**
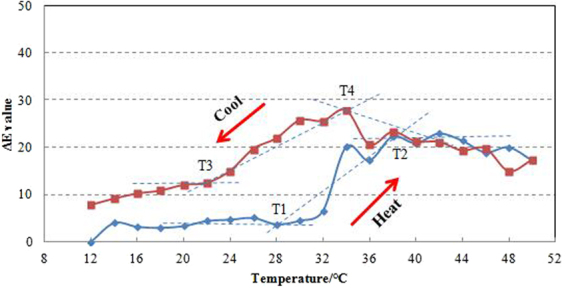
ΔE values of thermochromic wood veneers during heat and cool processing. T1 and T2 are the initial and final achromatic temperatures, while T4 and T3 are initial and final chromatic temperatures.

The color characteristics of thermochromic compounds depend on temperature and procedure are illustrated in Fig. 5.

T1 and T2 describe the initial and final achromatic temperatures during the decolorization procedure. As the temperature dropped below 28 °C, the color parameters (L, a, b) rarely changed, and the color change value ΔE tended toward stability. Between 28 °C and 38 °C, decolorization occurs. Combined with the results from Fig. 4, the color parameters (L, a, b) increased significantly. As the temperature rose above 38 °C, the decolorization slowed down. From the literature, it is known that reversible thermochromic change occurs via two competing reactions. At low temperature in a leuco dye–developer–solvent system, the solvent exists in its solid form. As the temperature increases, the solvent melts, and the leuco dye–developer system converts to a colorless state.

During the chromatic procedure, as the temperature increased above 34 °C, the color parameters a and b rarely changed, and the ΔL and ΔE values increased slightly as the temperature ranged from 50–34 °C. T4 and T3 described the initial and final chromatic temperatures during the reverse action. As the temperature ranged between 34–22 °C, the color parameters significantly decreased, the ΔE values increased, and the system regained color. Based on the color change characteristics, it can be deduced that the initial and final chromic temperatures were 34–22 °C during the reverse reaction. A perfect reversible process should return to the same color after cooling. As shown in Fig. 5, the color hysteresis phenomenon occurred between the heated and cooled states. This phenomenon was also described in previous studies^26^.

### Stability of the thermochromic properties

**Figure 6:**
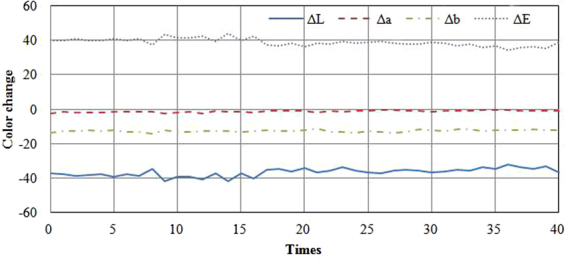
Color change curves vs time during cycling between 0 °C and 70 °C.

The stability of the thermochromic properties was evaluated according to the changes in colorimetric parameters in heat-cool loops. Figure 6 shows the colorimetric parameter values in 40 heat-cool loops. The ΔLn value is the difference between the initial value L1 and Ln after each loop, indicating Δa, Δb and ΔE.

As shown in Fig. 6, the Δa and Δb values rarely changed over 40 heat-cool loops. The ΔL (which ranged between −41.87 and −32.28) and ΔE values (which ranged between 34.46 and 43.81) fluctuated slightly during the loops, but the changes were quite small. Therefore, the heat-cool loop had no significant effect on the color stability of the thermochromic wood veneers, and the achromatic and chromatic sensitivities improved after first heat-cool loop.

### FTIR analysis of thermochromic compounds and veneers

**Figure 7:**
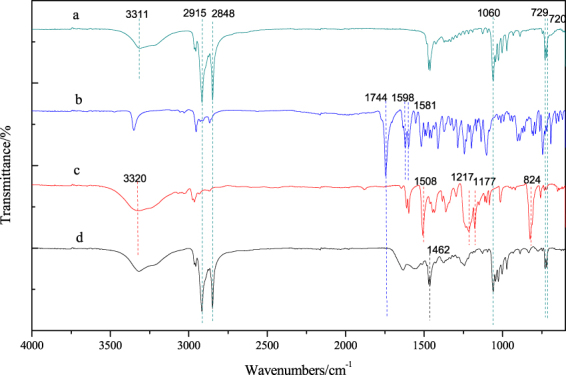
FTIR spectra of (**a**) 1-tetradecanol; (**b**) ODB-2; (**c**) bisphenol A; and (**d**) thermochromic compounds.

**Figure 8:**
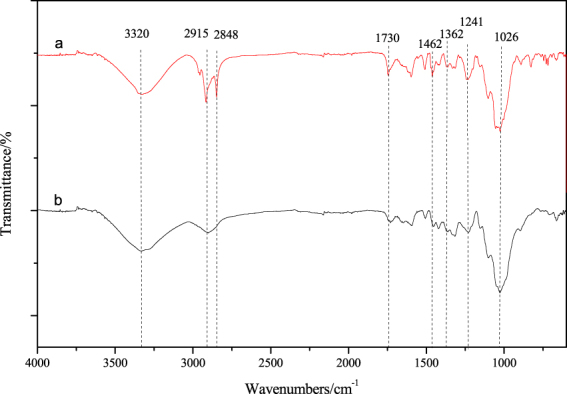
FTIR spectra of (**a**) thermochromic veneer and (**b**) control veneer.

Figures 7 and 8 show the FTIR spectra of the thermochromic compounds and wood veneers. The spectra illustrate the how well thermochromic compounds adhere to wood surfaces.

The spectra of thermomic compounds and pure compounds are shown in Fig. 7. The pure 1-tetradecanol exhibits the -OH stretching vibration at 3311 cm^−1^. The peaks at 2915 cm^−1^ and 2848 cm^−1^ belong to the -CH_2_ asymmetric stretching vibration of aliphatic chain. Similarly, the peaks at 729 cm^−1^ and 720 cm^−1^ are associated with -CH_2_ deformation. The strong peak appearing at 1060 cm^−1^ attributed to C-O stretching. The -OH stretching of pure bisphenol A has a maximum at 3320 cm^−1^. The C-C stretching in the aromatic ring of bisphenol A appeared at 1508 cm^−1^. The C-H deformation in aromatic ring is associated with the peak at 824 cm^−1^. The peak at 1217 cm^−1^ appearing in pure bisphenol A is associated with phenol OH deformation and the peak at 1177 cm^−1^ is associated with C-O stretching. ODB-2 dye belongs to a family of lactone compounds, and the typical spectral band appeared at 1740 cm^−1^–1725 cm^−1^ ^27^. In the FTIR spectra for pure ODB-2, three typical peaks at 1744 cm^−1^, 1598 cm^−1^ and 1581 cm^−1^ can be detected. The C=O band was noted at 1744 cm^−1^, and the C-C stretching of ODB-2 aromatic rings were represented by peaks at 1598 cm^−1^ and 1518 cm^−1^. In the spectra for the thermochromic compounds, the C=O band at 1744 cm^−1^ disappeared, and a new band appeared at 1462 cm^−1^. This band belongs to the -C-NH_2_ stretching band, confirming the existence of ODB-2 in the ring-open form.

Figure 8 represents the FTIR spectra of a control veneer and a thermochromic wood veneer. The control veneer showed bands at 3320 cm^−1^ (O-H stretching), 2914 cm^−1^ (-CH_2_ stretching), 1362 cm^−1^ (C-H stretching), and 1026 cm^−1^ (C-O stretching), which assigned to the characteristic peaks of cellulosic structure^28–30^. Compared with control veneer, thermochromic veneer has an obvious intense peaks appearing at 2915 cm^−1^ and 2848 cm^−1^, which attributed to the -CH_2_ stretching vibration of thermochromic compounds. Absorption band at 1730 cm^−1^ is stronger than that of control veneer, which was assigned to C=O stretching vibration of lacton ring in leuco dye. The peak at 1462 cm^−1^ (-C-NH_2_) indicating that the thermochromic compounds entered the interior of the wood veneers and successfully adhering into the wood. The intensity of the C-O stretching peak at 1026 cm^−1^ and the O-H vibration peak at 3320 cm^−1^ both increased, whereas and the wave number at 1241 cm^−1^ decreased, this might be considered as an covalent bond between wood fibers and thermochromic compounds.

## Conclusions

In this paper, a thermochromic wood veneer was fabricated. To achieve maximum ΔE values, the optimum thermochromic wood veneer can be fabricated by impregnating the wood with a suspension of thermochromic compounds (the ratio of ODB-2 to bisphenol A to 1-tetradecanol was 1:2:60) for 2.0 min at 65 °C. FTIR analysis demonstrated that intermolecular forces and covalent bond interaction formed between the thermochromic compounds and the wood. The color changed within a temperature range of 28–38 °C during the heating procedure, and the reverse action occurred within the temperature range of 34–22 °C. Color hysteresis was noted during the heating and cooling loops.

## Methods

### Materials

Juglans mandshurica veneers, 0.17 mm thickness, supplied by Taoshan Corporation (Harbin, China), were used for impregnation. The average moisture content was 7%. ODB-2 (purity 99.9%), bisphenol A (purity 99.9%) and 1-tetradecanol (purity 99.9%) were used for thermochromic compound synthesis and were supplied by Aladdin Chemicals Co., LTD (Shanghai, China).

### Experimental design

The orthogonal experimental design method is a highly efficient, fast, and economical experimental design method for evaluating the effects of various factors on material performances^31^. In this study, an orthogonal experimental design was adopted to select the optimum thermochromic compound formula and wood veneer impregnation parameters. To explore the optimum thermochromic compound formula, an orthogonal experiment with two factors, each at four levels, was conducted (Table 7).

**Table 7:** Factors and levels.

**Levels**	**Factors**	
	A (Mixing ratio of ODB-2 and bisphenol A)	B (Mixing ratio of ODB-2 and 1-tetradecanol)
1	1:1	1:20
2	1:2	1:40
3	1:3	1:60
4	1:4	1:80

Based on the optimum thermochromic compound formula, the effects of impregnation factors on wood veneer coloration were studied, and the various factors and selection levels are shown below (Table 8). Sixteen test sets were performed, with three repetitions for each set.

**Table 8:** Factors and levels.

Levels	Factors	
	Impregnation temperature/°C	Impregnation time/min
1	45	0.5
2	55	1.0
3	65	1.5
4	75	2.0

### Fabrication route

**Figure 9:**
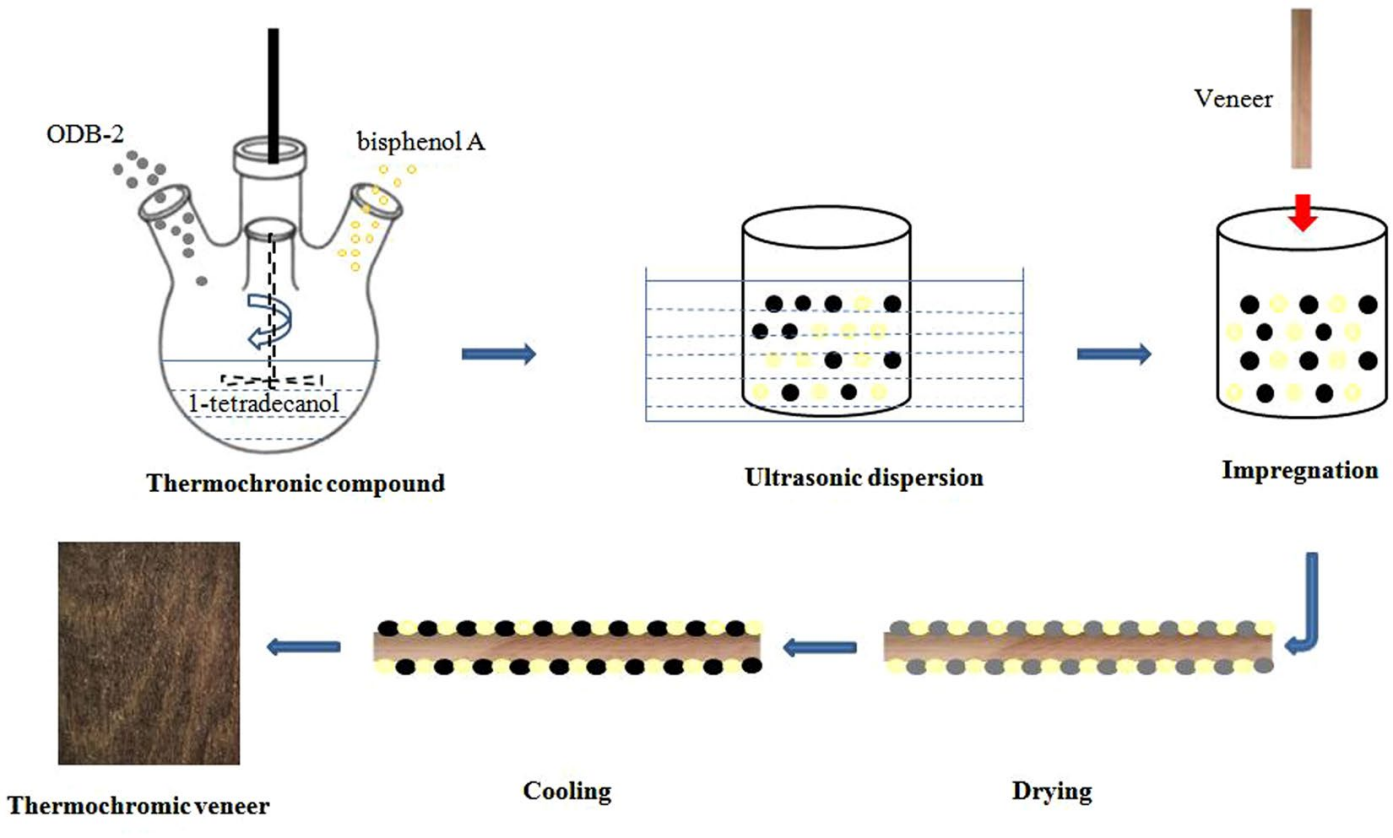
Schematic diagram for thermochromic wood veneer fabrication.

Predetermined amounts of ODB-2, bisphenol A and 1-tetradecanol were added to a boiling 4-neck flask and were heated in a 70 °C water bath. The component mixture was stirred with a Teflon paddle at 600 rpm/min for 1 hour. Reversible thermochromic compounds were obtained after natural cooling. The reversible thermochromic wood veneers were prepared by ultrasonic impregnation with a thermochromic compound suspension (Fig. 9). The wood veneer samples were treated in an ultrasonic bath and were then placed in an oven to dry for 40 min at 40 °C. After drying, the thermochromic veneer samples were conditioned in a consistent cabinet at 50% RH to equilibrium at temperatures set at 0–70 °C.

### Analysis of thermochromic properties

In this study, wood color was measured according to the CIElab system. The average color parameters, including L (lightness index), a (red-green index) and b (yellow-blue index) of the wood veneer surface were measured using an NP10QC chroma meter (3NH, Inc., China).

The color difference value ΔE was calculated using the following equation:

1 $${\rm{\Delta }}E=\sqrt{{\rm{\Delta }}{L}^{2}+{\rm{\Delta }}{a}^{2}+{\rm{\Delta }}{b}^{2}}$$

Fourier transform infrared (FTIR) spectra were recorded using a Nicolette 6700 (Nicolet, USA). The statistical analyses were performed using SPSS statistics 18.0 (IBM Corporation, USA) software. Range and ANOVA analyses were conducted to illustrate the effects of different factors on the color parameters.

## Electronic supplementary material

Dataset 1
